# Correlation of Chronic Cervico-Cranio-Mandibular Pain in Individuals with Adverse Childhood Events: An Observational Study

**DOI:** 10.3390/healthcare12212118

**Published:** 2024-10-24

**Authors:** Iván Ruiz-Rodríguez, María Dolores Sosa-Reina, David Ruiz-Zaragoza, Valentina Vargas-Sánchez, Álvaro Fernández-Martínez, Rubén López-Bueno, Carlos Romero-Morales, Jorge Hugo Villafañe

**Affiliations:** 1 Department of Physiotherapy, Faculty of Sport Sciences, Universidad Europea de Madrid, 28670 Villaviciosa de Odón, Spain; ivan.ruiz.rodriguez@gmail.com (I.R.-R.); ruizzaragozadavid793@gmail.com (D.R.-Z.); alvarofernandez004@gmail.com (Á.F.-M.); carlos.romero@universidadeuropea.es (C.R.-M.); mail@villafane.it (J.H.V.); 2 Musculoskeletal Pain and Motor Control Research Group, Faculty of Sport Sciences, Universidad Europea de Madrid, 28670 Villaviciosa de Odón, Spain; valvar97@hotmail.com; 3 Department of Physical Medicine and Nursing, University of Zaragoza, 50009 Zaragoza, Spain; rlopezbu@unizar.es; 4 National Research Centre for the Working Environment, 2100 Copenhagen, Denmark; 5 Exercise Intervention for Health Research Group (EXINH-RG), Department of Physiotherapy, University of Valencia, 46010 Valencia, Spain

**Keywords:** chronic pain, headache, mandibular, adverse childhood events

## Abstract

Objectives: This cross-sectional observational study examines the relationship between chronic cervico-cranio-mandibular pain, a significant health concern associated with temporomandibular disorders, and adverse childhood experiences (ACEs). Given the high prevalence of cervical pain and the gap in adequate treatment for temporomandibular disorders, this research highlights the interplay between psychological, social factors, and musculoskeletal health. Methods: The study, conducted from January to June 2023, included 114 participants (mean age = 31 ± 12 years, 69.3% female) experiencing chronic cervico-cranio-mandibular pain. Pain severity and dysfunction were assessed, and exposure to ACEs was measured using validated questionnaires. Statistical analysis, performed using Jamovi (v 2.23.28) software. Results: Data revealed a significant correlation between the number of ACEs and both pain intensity (r = 0.254, *p* = 0.006, η^2^ = 0.062) and disability (r = 0.262, *p* = 0.005, η^2^ = 0.068). However, no significant association was found between ACEs and mandibular functional limitation (*p* = 0.222). These findings suggest that while early life stressors impact overall health, their specific influence on cervico-cranio-mandibular pain is limited. Conclusion: The study emphasizes the importance of integrated early intervention strategies to mitigate the long-term musculoskeletal repercussions of adverse events, advocating for comprehensive mental health support and preventive measures. This research contributes valuable insights into the necessity of a multifaceted approach to understanding, diagnosing, and treating musculoskeletal disorders, highlighting the complexity of their causes and effects.

## 1. Introduction

Chronic cervico-cranio-mandibular (CCM) pain is a prevalent musculoskeletal disorder, with significant global impact. Despite consistent clinical presentations from 1990 to 2019, it remains the fourth leading cause of global disability, affecting approximately 70% of individuals at some point in their lives [1]. Chronic forms of this condition develop in 30–50% of cases, with an annual incidence of 10–21%. CCM pain is closely associated with temporomandibular disorders (TMD), which complicates diagnosis and treatment due to their shared musculoskeletal involvement. It is estimated that 60–70% of the global population experiences TMD symptoms, with women being almost twice as affected as men [2].

TMD manifests through a variety of clinical symptoms, including joint noise, bruxism, facial pain, joint and muscle sensitivity, and migraines [3,4,5]. Despite this high prevalence, only a small proportion (5%) of affected individuals receive adequate treatment [6,7]. Additionally, TMD has been linked to disruptions in the central nervous system (CNS), manifesting as psychopathological symptoms, circadian rhythm dysregulation, and autonomic nervous system imbalances. These factors result in altered activity in key brain regions such as the hippocampus, amygdala, and hypothalamus [2].

The complexity of diagnosing and treating cervico-cranio-mandibular disorders is compounded by psychological and social factors. Previous studies, such as that of Grossi et al. [8], identified physical, emotional, and sexual abuse as significant risk factors for developing TMD. Other research has demonstrated associations between self-reported TMD symptoms and experiences of violence or abuse [9,10]. Additionally, the COVID-19 pandemic has led to an increased prevalence of TMD and bruxism, driven by elevated psychosocial stressors such as anxiety and depression [11]. CCM pain extends beyond its physical manifestations, severely affecting social functioning by fostering social isolation and exacerbating psychological distress. Moreover, evidence suggests that the presence of myofascial trigger points, particularly in the neck and shoulder muscles, is common in musculoskeletal pain syndromes like CCM pain, perpetuating sensitization and pain chronicity, which further complicates treatment outcomes [12].

In addition, cervico-cranio-mandibular pain can have a profound impact on individuals’ social lives, hindering interpersonal relationships by limiting their participation in social and family activities. Patients suffering from CCM pain often experience depressive symptoms, hypervigilance, stress, and sleep disturbances, among others, which negatively affect their quality of life [13]. As persistent pain interferes with these key areas, individuals are likely to become socially isolated, which in turn can intensify stress and anxiety, creating a vicious cycle that perpetuates both pain and emotional and social difficulties.

Adverse childhood experiences (ACEs), defined as traumatic or stressful events occurring before the age of 18, have also been implicated in the development of chronic pain conditions. The prevalence of ACEs in Europe is 23.5% for individuals reporting one ACE and 18.7% for those reporting two or more, with higher figures reported in North America [14]. ACEs encompass abuse (physical, emotional, sexual), neglect, and household dysfunction. Bullying, which affects 28–32% of the population, is also considered a significant ACE [15]. Exposure to these adversities has been shown to increase the risk of developing chronic health conditions, such as headaches, depression, and anxiety, later in life [16,17].

Physical and sexual abuse during childhood, with prevalence rates of 22.9% and 9.6%, respectively, are significantly associated with the chronicity and severity of pain in adulthood [18]. These findings suggest that early life stressors have long-lasting effects on both psychological and physical health, particularly in relation to the development of chronic pain.

This study aims to investigate the role of ACEs as a primary risk factor for the chronicity of cervico-cranio-mandibular pain, examining both psychological and physical dimensions of the disorder. We hypothesize that individuals with a greater number of ACEs will exhibit increased pain intensity and disability related to CCM pain, consistent with prior evidence linking early life stressors to chronic pain. Additionally, it is anticipated that the relationship between ACEs and CCM pain may vary across functional domains, with a stronger association expected for pain intensity and disability compared to mandibular functional limitation. This reflects the complex interaction between psychological trauma and physical health outcomes.

This research contributes a novel perspective by exploring the relationship between ACEs and CCM pain in a contemporary European population, focusing on how ACEs differentially impact mandibular dysfunction and neck functional limitation. By utilizing recent data, this study offers an updated view on the importance of preventive strategies to mitigate the long-term consequences of early life stressors on physical health.

## 2. Materials and Methods

This descriptive, observational cross-sectional study received approval from the European University Research Commission (CIPI/23.140 on 13 April 2023), and all participants provided informed consent prior to their involvement. Recruitment was carried out using a “snowball” sampling method, both online and in person, at the European University of Madrid and various health centers, targeting individuals aged 18–60 with chronic cervico-cranio-mandibular pain. All procedures were conducted in accordance with the Declaration of Helsinki, and informed consent was obtained from each participant.

Inclusion criteria were: (1) subjects aged between 18 and 60 years with chronic pain and/or persistent pain lasting more than three months in the cervico-cranio-mandibular region, and (2) have expressed their willingness through the signing of informed consent. Chronic cervico-cranio-mandibular pain was defined as persistent pain lasting more than three months in the cervico-cranio-mandibular region, aligning with the study’s inclusion criteria. While secondary outcome measures, including the Graded Chronic Pain Scale (GCPS), Neck Disability Index (NDI), and Jaw Functional Limitation Scale (JFLS-8), were used to assess pain intensity and functional limitations, the primary inclusion criterion was self-reported pain duration rather than specific cutoff scores. These validated instruments ensured consistency and supported the diagnosis of chronic pain, following established guidelines in chronic musculoskeletal research. 

As exclusion criteria, (1) individuals with previous surgery in the cervico-cranio-mandibular region were not included in the study or (2) subjects who did not provide informed consent.

All participants completed a survey designed in Google Forms where they provided initial data and consented to participate in the study. Subsequently, participants completed a series of self-administered validated Spanish questionnaires such as the chronic pain grading scale questionnaire, NDI, JFLS-8 (Jaw Functional Limitation Scale), ACEs (Adverse Childhood Experiences), and MIDAS questionnaire (Migraine Disability Assessment) via Google Forms. These questionnaires provided information on chronic pain in the cervico-cranio-mandibular sphere and adverse events in childhood.

### 2.1. Measurements

#### 2.1.1. Chronic Pain Magnitude

The Spanish version of the GCPS was used as a measure for chronic pain, as it is a valid tool in Spain for anticipating chronic pain. This tool is utilized to assess both pain intensity and the disability associated with it. The scale consists of 8 items that evaluate the patient’s pain over the past 6 months. It ranges from grade 0 (no pain) to grade 4 (completely limiting), with a 95% confidence interval. This tool can assess any type of chronic pain, as it is not specific to any particular pathology. In this study, the questions were related to cervico-cranio-mandibular pain [19].

#### 2.1.2. Adverse Events at Childhood

Adverse Childhood Experiences Questionnaire in Spanish (EAI) is a self-reported questionnaire, with three main areas: The ACEs questionnaire divides childhood trauma into three main categories: (1) abuse, which includes psychological, physical, and sexual abuse; (2) neglect, which can be physical or emotional; and (3) family dysfunction. Sexual abuse is categorized solely under “sexual abuse” and is not further sub-categorized into other forms of abuse such as neglect, psychological, or physical abuse. The CI of this questionnaire is 84% [20].

### 2.2. Secondary Outcomes

Cervico-cranio-mandibular pain cannot be measured generically, but rather each part has to be measured separately, as there is not any questionnaire for it. The secondary outcomes analyzed were cervical function limitation, mandibular function limitation, and disability due to headache.

#### 2.2.1. Cervical Functional Limitation

The Neck Disability Index (NDI) is a self-administered questionnaire that measures functional limitation caused by neck discomfort and how it affects work-related daily living activities (concentrating, lifting, working) and non-work-related activities (reading, personal care, driving, sleep, and leisure), as well as the intensity of self-reported pain (headache, pain) [21]. There are 10 sections, with 6 possible answers with progressive levels of ability. The scores range from 0–5 for each of 10 items, with a maximum of 50 points with a 98% CI [21].

#### 2.2.2. Mandibular Function Limitation

The JFLS-8 Mandibular Function Scale was used for measuring limiting function on the mandibular mobility, chewing verbal and emotional expression, and connecting pain and limitation in the facial and craniomandibular area. It consists of 8 questions that have to be answered from 0 (no limitation) to 10 (severe limitation), with a 82% CI [22].

#### 2.2.3. Disability Due to Headache

The Migraine Disability Assessment (MIDAS) Questionnaire evaluates the degree of disability related to migraine. The scale consists of 7 items; the first 5 are based on daily living activity restriction, and the last 2 are based more on the frequency and intensity of headache. It has a 95% CI [23].

### 2.3. Procedure

All participants filled out a Google Forms survey, providing the initial data and consent to participate in the study. After this, participants entered through a link to the questionnaires mentioned above, to be answered in the time the person would consider prudent. These questionnaires allowed us to collect information on chronic pain in the cervico-cranio-mandibular sphere and adverse events in childhood. The results obtained were reviewed, and the corresponding variables were compared to obtain the results.

### 2.4. Statistical Analysis

The statistical analysis was conducted using SPSS version 28.0 software (SPSS Inc., Chicago, IL, USA). A confidence level of 95% (*p* < 0.05) was established for the interpretation of statistical tests. The normality of quantitative variables was assessed using the Shapiro–Wilk test. Correlations were performed using Spearman’s rho test. For categorical variables, odds ratios and chi-square tests (with contingency tables of 2 × 2, 2 × 3, and 4 × 2) were calculated. Additionally, in descriptive statistics, absolute and relative frequencies (percentage) were presented for categorical variables, and mean and standard deviation were presented for normally distributed quantitative variables, while median and interquartile range were presented for non-normally distributed variables.

## 3. Results

### 3.1. Participants’ Demographic and Clinical Data

The study included 114 participants, of whom 52.63% (60 participants) reported experiencing adverse childhood events (adverse events group). The sample was predominantly female (79 females; 69.29%), with an average age of 60 ± 18 years, as it is described in Table 1. The variables analyzed were mandibular function limitation (MFL), neck functional limitation (NDI), adverse childhood events, pain intensity, headache disability, as well as pain-related disability.

### 3.2. Primary Outcomes

There was a significant association between pain intensity and MIDAS (*p* = <0.001); pain intensity and NDI (*p* = <0.001); pain disability and MIDAS (*p* = <0.001); and disability with NDI (*p* = <0.001). There was no association between pain intensity and mandibular function limitation (*p* = 0.222); pain intensity and adverse events (*p* = 0.055); pain disability and mandibular function limitation (*p* = 0.359); and pain disability and adverse events (*p* = 0.211).

#### Chronic Pain and Adverse Childhood Events

EAI and graded chronic pain scales were applied. We can observe that both pain intensity and pain disability are correlated with adverse childhood events (Table 2).

### 3.3. Secondary Outcomes

#### 3.3.1. Mandibular Function and Headache Disability

The JFLS-8 Mandibular Function Scale and MIDAS Questionnaire were used for measuring the association between mandibular dysfunction and headache disability. A significant correlation was found between mandibular function limitation and headache-related disability (*p* < 0.001), indicating that greater mandibular limitation is associated with more severe headache disability and frequency.

#### 3.3.2. Mandibular Function and Pain Intensity and Pain Disability

The JFLS-8 Mandibular Function Scale and GCP Scale were used. A significant correlation was found between mandibular function limitation and pain intensity, indicating that greater mandibular limitation is also correlated with pain intensity. Additionally, a significant correlation was also observed between pain disability and the limitation of mandibular function.

#### 3.3.3. Adverse Childhood Events, Mandibular Function and Headache Disability

The JFLS-8, MIDAS Questionnaire, and EAI were used to match up results. Significant correlation was found between adverse childhood events and mandibular function limitation, as well as between adverse childhood events and headache disability.

#### 3.3.4. Neck Functional Limitation and Mandibular Function

The JFLS-8 and NDI Questionnaires were used for measuring associations. A significant correlation was also observed between neck functional limitation and substantial mandibular function restriction, suggesting that neck limitations are linked to more significant restrictions in mandibular function.

#### 3.3.5. Neck Functional Limitation and Adverse Childhood Events

The NDI Questionnaire and ACE Scale were used for measuring correlation and associations. A significant correlation was found between neck limitations and more adverse events.

## 4. Discussion

The primary objective of this study was to determine whether ACEs constitute a risk factor for the development of CCM pain and to assess if an increased number of ACEs correlates with heightened pain intensity and disability in adulthood. Rather than employing a dichotomous comparison between individuals with and without ACEs, correlation analyses were conducted to investigate whether the number of ACEs had a graded association with both pain intensity and disability. This approach avoids oversimplifying the relationship, captures gradual associations more accurately, increases statistical power, and aligns with previous literature that shows cumulative ACEs are linked to worse health outcomes [24]. The results demonstrated a statistically significant correlation between the number of ACEs and pain intensity (r = 0.254, *p* = 0.006), disability (r = 0.262, *p* = 0.005), mandibular function (r = 0.240, *p* = 0.01), headache disability (r = 0.306, *p* < 0.001), and neck functional limitation (r = 0.282, *p* = 0.02). These findings suggest that a greater accumulation of childhood adverse events is associated with a more severe chronicity of CCM pain. Additionally, these results also indicate that ACEs impact multiple dimensions of physical health, including mandibular function and functional limitations of the neck and head, reinforcing the need for multidimensional treatment approaches.

These findings are consistent with the existing literature. For instance, Goldberg et al. (1999) [25] indicated that any form of adverse childhood experience can substantially impact family equilibrium and long-term health outcomes. Approximately 40% of individuals suffering from chronic pain report having experienced at least one adverse event during childhood, reinforcing the necessity of considering early life experiences when managing chronic pain conditions. Additionally, Eitne et al. (2009) [26] reported that patients with temporomandibular pain had a higher likelihood of having undergone traumatic events before the age of 16, thereby increasing their risk of developing chronic pain and disability later in life.

Furthermore, research by Wolke et al. (2015) [15] and Macedo et al. (2019) [27] demonstrated how early life stress/bullying is a risk factor for inflammatory problems and associated health issues, which can persist into adulthood and thereby increase the comorbidity between depression and chronic pain. Recent research, such as the study by Giannouli and Tsolaki (2023) [28,29], further emphasizes the role of stressful life events in cognitive performance and health outcomes in older adults and Alzheimer’s patients. While stressful life events did not directly impact cognitive decline or financial capacity in Alzheimer’s patients, their cumulative effect was significant, aligning with our findings that ACEs contribute to chronic CCM pain. Both studies suggest that early interventions targeting stress management may mitigate some of the detrimental effects of these experiences.

Despite the lack of a statistically significant correlation between ACEs and mandibular functional limitation in this study, previous literature, such as the work by Grossi et al. (2018) [8], underscores the need to evaluate physical, sexual, and emotional abuse in patients with temporomandibular disorders, particularly those who exhibit poor treatment responses. Similarly, Morales-Salazar et al. (2022) [30] highlighted the relationship between altered dentofacial characteristics and the prevalence of childhood bullying, with adverse impacts on quality of life, emotional stability, self-esteem, and academic performance.

These observations emphasize the interconnectedness of chronic pain elements in the cervico-cranio-mandibular region—such as limitations in mandibular function, headache-related disability, and neck functionality—with ACEs. Failing to account for these factors in clinical evaluations could result in a superficial understanding of patient presentations, potentially leading to suboptimal treatment outcomes. Moreover, the cumulative effect of ACEs may be a contributing factor in the development of chronic CCM pain, with the severity of pain and associated disability increasing alongside the number of adverse events experienced.

In addition to the primary findings, we conducted exploratory analyses to investigate whether there were any differences based on gender and age. Although our sample was predominantly female (69.29%), no statistically significant gender-based differences in pain intensity or disability were found. Similarly, no significant variations were observed when stratifying the data by age groups. Nonetheless, the trends observed suggest potential gender differences in pain intensity and disability that warrant further investigation in future research with larger and more diverse samples. It would also be beneficial to explore other demographic factors, such as socioeconomic status and education, which may influence the relationship between ACEs and chronic pain [31].

### 4.1. Implications for Rehabilitation

The results of this study point to a critical need for integrated rehabilitation strategies that commence early in life to mitigate the long-term musculoskeletal consequences of ACEs. Given the established correlation between ACEs and chronic cervico-cranio-mandibular pain, rehabilitation professionals should consider early, proactive interventions. These interventions should not only address the physical manifestations of pain but also incorporate psychological support to handle the emotional aftermath of such adverse experiences. Multidisciplinary approaches involving physiotherapists, psychologists, and pediatric specialists are essential to providing a comprehensive treatment plan. This strategy ensures that interventions are not merely reactive but preventive, aiming to reduce the incidence of chronic pain and associated disabilities later in life.

Furthermore, this study highlights the importance of educational programs that train caregivers and educators to recognize and respond to the signs of stress and trauma in children, thereby fostering a supportive environment that can prevent the progression of ACEs to chronic physical conditions. Future research should focus on longitudinal studies that examine the trajectory of pain and disability in individuals with ACEs over time, as well as the biological mechanisms (e.g., neuroendocrine and inflammatory pathways) that mediate the relationship between ACEs and chronic pain. Additionally, exploring resilience factors, such as social support and coping strategies, could further enhance intervention approaches, providing more personalized and effective treatment plans.

### 4.2. Study Limitations

This study presents several limitations that are important to consider when interpreting its findings. The absence of a control group constitutes a notable limitation. Due to the exploratory nature of this research and challenges in recruitment, we were unable to include a comparable cohort of individuals without cervico-cranio-mandibular pain. This omission may limit the generalizability of the findings and restrict the ability to fully assess the influence of ACEs in comparison to individuals not affected by this condition. Future research should address this limitation by incorporating a control group, which would enable more robust comparisons and enhance the validity of the results.

Additionally, the sample size, while adequate for an exploratory analysis, may not provide the statistical power necessary to detect smaller effect sizes or allow for detailed subgroup analyses. As a result, some potentially important relationships between ACEs and various outcomes, such as functional limitations or other demographic factors, may have gone undetected. A larger sample size in future studies would strengthen the ability to draw more definitive conclusions and allow for a more nuanced exploration of how factors such as sleep dysfunction, catastrophizing, socioeconomic status, and ethnicity interact with chronic cervico-cranio-mandibular pain.

The reliance on self-reported data to define chronic pain also introduces the potential for recall and reporting biases. Although validated instruments were employed to assess pain intensity and functional limitations, the subjective nature of these reports could influence the study’s outcomes. Furthermore, while the use of standardized questionnaires helps to mitigate variability, the self-reported nature of ACEs and pain could still contribute to inconsistencies in data quality.

Lastly, the cross-sectional design of this study limits the ability to infer causality between ACEs and chronic cervico-cranio-mandibular pain. Longitudinal studies are needed to better understand the temporal relationship between early life stressors and the development or persistence of chronic pain in adulthood. Despite these limitations, the study provides valuable preliminary insights into the complex relationship between ACEs and musculoskeletal health, underscoring the need for more comprehensive, controlled, and longitudinal research.

## 5. Conclusions

In conclusion, a weak to very weak association was identified between adverse childhood events and the presence of pain, disability, mandibular function, headache disability, and neck functional limitation. Future research should prioritize multidisciplinary interventions to address the long-term effects of ACEs more effectively.

## Figures and Tables

**Table 1:** Demographic data of participants.

Characteristic	Description
Total number of participants	114
Percentage of participants with adverse childhood events	52.63%
Gender	69.29% female
Average age	31 ± 12 years

**Table 2:** Significant correlation between key variables.

Variables Compared	Questionnaires Applied	Correlation Coefficient	*p*-Value	Interpretation
Pain intensity and adverse childhood events	EAI, GCPS	0.254	0.006	There is a correlation between pain intensity and adverse childhood events.
Disability and adverse childhood events	EAI, GCPS	0.262	0.005	There is a correlation between disability and adverse childhood events.
Mandibular function and headache disability	JFLS-8, MIDAS	0.340	<0.001	Greater mandibular limitation is correlated with more severe headache disability.
Mandibular function and pain intensity	GCPS, JFLS-8	0.349	<0.001	Greater mandibular limitation is also correlated with pain intensity.
Mandibular function and disability	GCPS, JFLS-8	0.418	<0.001	Greater mandibular limitation is also correlated with disability.
Adverse childhood events and mandibular function	AEI, JFLS-8	0.240	0.01	Adverse childhood events and mandibular limitations are correlated.
Adverse childhood events and headache disability	AEI, MIDAS	0.306	<0.001	Adverse childhood events and headache disability are correlated.
Neck functional limitation and mandibular function	NDI, JFLS-8	0.461	<0.001	Neck limitations are linked to more significant restrictions in mandibular function.
Adverse childhood events vs neck functional limitation	EAI, NDI	0.282	0.002	There is a correlation between having had adverse events with more neck limitation.

## Data Availability

Data generated during the study are available upon request from the corresponding author.
